# Termite Diversity in Ecuador: A Comparison of Two Primary Forest National Parks

**DOI:** 10.1093/jisesa/iez129

**Published:** 2020-01-09

**Authors:** Cecilia A L Dahlsjö, Cynthia S Valladares Romero, Carlos-Iván Espinosa Iñiguez

**Affiliations:** 1 Environmental Change Institute, School of Geography and the Environment, University of Oxford, South Parks Road, United Kingdom; 2 Departamento de Ciencias Biológicas, Universidad Técnica Particular de Loja, Loja, Ecuador

**Keywords:** abundance, Ecuador, diversity, termite, species richness

## Abstract

Termites are one of the key ecosystem engineers in tropical forests where they play a major role in decomposition rates, both above and belowground. The interest in termite ecology and biogeography has increased in the last few decades; however, the lack of comparable data has limited the wider impact of termite research. For Ecuador, termite studies are relatively rare and comparable data that are collected using standardized sampling methods are missing. In this study, we aim to 1) provide comparable data of termite species and feeding-group diversity from two primary forests in Ecuador and 2) explore the differences in termite species and feeding-group diversity between the two forest sites. Sampling took place in the national parks of Yasuní and Podocarpus where three belt transects (100 × 2 m) following Jones and Eggleton (2000) were conducted in each forest. We found that termite species richness was higher in Yasuní (56 species) than in Podocarpus (24 species) and that 57% of the sampled termite genera had never previously been recorded in Ecuador. The inter-site species dissimilarity was almost complete (Bray Curtis (±SD), 0.91 ± 0.01), which may have been linked to the difference in tree density and species richness in the two forests. Termite feeding-groups diversity was significantly higher in Yasuní than in Podocarpus with the exception of soil-feeding termites which may have been due to competition between humus- and soil-feeding species.

Forest ecosystem functioning is primarily driven by microorganisms and invertebrate decomposers that contribute to the breakdown of organic matter and nutrient cycling (Brussaard et al. 1997). In tropical forests, termites have been shown to play a major role in decomposition rates, both above (Vasconcellos and Moura 2010) and belowground (Dahlsjö et al. 2014a), as well as contributing to soil productivity (Ayuke et al. 2011), plant growth (Jouquet et al. 2011), and habitat stability (Jouquet et al. 2014). Their importance is probably due to their high abundance and biomass, particularly in undisturbed lowland tropical forests (Bignell and Eggleton 2000), and their ability to break down a range of organic matter aided by symbiotic gut microorganisms (Hongoh and Ohkuma 2010) and external fungi (Aanen and Eggleton 2005).

Better understanding of the importance of termites in ecosystem functioning has created a heightened interest in their ecology and biogeography, which has meant an increase in termite studies in the last few decades (see Šobotník and Dahlsjö (2017) for a review). However, termite studies are still not as abundant as those of other decomposers (536 termite studies compared with 765 earthworm studies in 2018, Web of Knowledge 2019), and pale in comparison with other eusocial insects (344,746 ant and 512,312 bee studies in 2018, Web of Knowledge 2019). Further, the increased focus on model-based approaches require comparable baseline data, the former of which paradoxically reduce the impact of descriptive studies. This is particularly a dilemma in termite ecology and biogeography where only limited conclusions can be drawn due to the lack of comparable data (Bignell and Eggleton 2000, Eggleton 2000, Davies et al. 2003, Dahlsjö et al. 2014b).

For Ecuador, termite studies are relatively rare and data that are collected using standardized sampling methods are missing. Bahder et al. (2009) provide the most up-to-date species list for Ecuador, including a total of 72 termite species and morphospecies from the Amazon to the Andes. While the list is an important contribution to termite research, the data are not comparable, due to the nonstandardized sampling and lack of information about the morphospecies, and the low representation of soldierless species (*Anoplotermes-*group) (eight species compared with 40 species encountered in an undisturbed lowland rain forest in Peru; Dahlsjö et al. 2014a).

Termite species richness has been shown to decline with elevation with soil-feeding termites disappearing at a faster rate than wood-feeding species (Palin et al. 2010). The faster decline of soil-feeders may be due to their lower energy food substrate and the reduction of metabolic rate with reduced temperatures (Eggleton et al. 1998, Nalepa 2011, Dahlsjö et al. 2015). While wood-feeding termites tend to be more tolerant to arid environments and variable microclimatic conditions, no unique species have been found in high-altitude forests suggesting that there are no elevation specialists among termites (Jones et al. 2003, Palin et al. 2010). Termites are also sensitive to disturbance of the canopy, which increases temperature and moisture variability and resource availability such as fallen dead wood (Eggleton et al. 1996, Davies 2002).

In this study, we aim to 1) provide comparable data of termite species and feeding-group diversity from two primary forest national parks in Ecuador using a standardized transect method and 2) explore the differences in species and feeding-group diversity between the two forest sites. Specifically, we predict that 1) Yasuní will have higher species richness and intra-site variability than Podocarpus due to the lower elevation and higher tree diversity (Jones 2000, Gathorne-Hardy et al. 2001, Palin et al. 2010), 2) termite species composition in the two forests will overlap due to the lack of elevation specialists (Jones et al. 2003, Palin et al. 2010), 3) the proportion of wood- versus soil-feeding and foraging termites will be higher in Podocarpus than in Yasuní due to the rapid decline of the latter with elevation (Eggleton et al. 1998, Gathorne-Hardy et al. 2001), and 4) environmental variables including canopy cover and leaf- and woody litter will be associated with termite feeding-group species diversity due to their response to canopy disturbance (Eggleton et al. 1996) and resource availability (Davies 2002). Additionally, techniques that enable the identification of soldierless species, with the help of internal gut morphology (Fontes 1987, Bourguignon et al. 2016), are expected to unearth species that have not yet been recorded in Ecuador.

## Materials and Methods

### Study Site

Sampling took place in Yasuní National Park (0°40′30.48″S, 76°23′58.84″W) and Podocarpus National Park (4°7′6.29″S, 78°58′3.11″W) on the eastern side of the Andes in Ecuador in August 2016 during the dry season (Supp Fig. S1 [online only]). Yasuní is a lowland (~ 200 m above sea level) evergreen primary forest that covers an area of 9,820 km^2^ (Finer et al. 2009) and is situated in north-east Ecuador, close to the Peruvian border. Yasuní National Park supports 1,017 tree species with average tree density and tree basal area of 654 trees ha^−1^ and 30.2 m^2^ ha^−1^, respectively (Pitman et al. 2002). The Yasuní region has mean annual rainfall and temperature of 2,826 mm and 28°C, respectively (Valencia et al. 2004) (see Table 1 for transect specific information). The park was founded in 1979 (Finer et al. 2009) and is considered one of the primary biodiversity hotspots on Earth (Pitman et al. 2002, Bass et al. 2010). As well as high biodiversity, the ground that Yasuní was founded on is rich in oil and is said to harbor 20% of the oil reserves in Ecuador (Martin 2011, Espinosa 2013), the exploitation of which may conflict with conservation interests.

**Table 1.** Transect specific variables for the two study sites Yasuní National Park and Podocarpus National Park

	Yasuní National Park			Podocarpus National Park		
Transect	T1	T2	T3	T1	T2	T3
Latitude	0°40′30.48″S	0°41′0.03″S	0°40′33.93″S	4°7′6.39″S	4°7′0.16″S	4°7′5.35″S
Longitude	76°23′58.84″W	76°23′0.01″W	76°23′53.05″W	78°58′3.11″W	78°58′9.74″W	78°58′14.91″W
Elevation (m)	219	208	208	1,074	1,029	1,044
Canopy cover (%)	91.4 ± 4.4	88.9 ± 5.2	92.7 ± 3.7	89.8 ± 4.2	90.2 ± 3.6	88.8 ± 2.7
Leafy litter weight (g)/section (10 m^2^)	52.9 ± 49.8	57.8 ± 63.0	99.9 ± 64.6	375.9 ± 160.9	247.1 ± 70.8	233.5 ± 80.4
Woody litter weight (g)/section (10 m^2^)	351.5 ± 167.2	685.9 ± 343.2	376.4 ± 85.5	45.4 + 95.6	16.6 ± 14.6	9.6 ± 12.0

The ± provides standard error information.

Podocarpus National Park is an evergreen low montane primary forest that is situated on the slopes of the Andes in southern Ecuador. Podocarpus National Park harbors 75 tree species with an average tree density and basal area of 748 trees ha^−1^ and 33.4 m^2^ ha^−1^, respectively (Krashevska et al. 2007, Bendix et al. 2013). The study site was located at an elevation of 1,000 m and has mean annual rainfall and temperature of 1,250 mm and 20°C, respectively (Podocarpus National Park 2018) (see Table 1 for more site-specific information). The national park was founded in 1982 and covers an area of 1,450 km^2^ and is known for its high diversity of bird species, which has spawned a growing tourist industry.

### Sampling and Identification

In each of the two study sites, three standardized belt transects (100 × 2 m) following Jones and Eggleton (2000) were conducted and located at least 200 m apart. Each transect was split into twenty 10 m^2^ sections (2 × 5 m) in which termites were collected from a range of possible microhabitats following those specified in Jones and Eggleton (2000) including leaf litter, deadwood (logs and branches), runways, termite mounds, at the base of plants, and in twelve 12 × 12 × 10 cm soil pits. In each 10 m^2^ section of the transect, the search protocol was one person sampling for an hour (or two people sampling for 30 min each).

Canopy cover was measured in each 10 m^2^ transect section with a camera positioned on the ground perpendicular to the canopy. The photographs were taken with a fisheye lens and analyzed using ImageJ. The photographs were black and white transformed, and the percentage of canopy cover calculated using the equation: *Canopy cover* (%) = *C*/(*C* + *S*) * 100 where *C* is the pixels of canopy cover and *S* is the pixels of sky in the image. Leaf and woody litter were collected from five sections (10 m^2^ each) in each transect, specifically sections 1, 5, 10, 15, and 20, once termite sampling had been conducted. The two litter types were dried to constant weight. The elevation and latitudinal and longitudinal coordinates were recorded at the transect level with a GPS.

Termites were identified to genus, and where possible to species using Constantino (2002), Bourguignon and Roisin (2011), and the reference collection at the Natural History Museum (NHM) in London. For specimens of soldierless forms, or where soldiers could not be found, it was necessary to determine gut and enteric valve morphologies following Pinzon et al. (2019) and Bourguignon et al. (2010, 2016) and the key to the Apicotermitinae of the Guiana Shield (L. M. Hernandez, unpublished data). Where existing keys were inadequate or absent, specimens were assigned to morphospecies following Dahlsjö et al. (2014a) (see Supp Table S1 and Supp Fig. S2 [online only] for a full species list and enteric valve images of the soldierless *Anoplotermes*-group morphospecies).

Five termite feeding-groups are universally recognized (Martius 1994, Donovan et al. 2001, Inward et al. 2007) as well as a few minor groups (e.g., Martius et al. 2000). All major feeding-groups, with the exception of fungus-growing termites (Aanen and Eggleton 2005), inhabit South America namely: feeding-group I (FGI) that feeds on sound wood and comprises all non-Termitidae species, FGII that feeds on wood and leaf litter (Termitidae), FGIII that feeds on humus with visible plant particles (Termitidae), and FGIV that feeds on soil without visible plant particles (Termitidae).

### Data Analysis

Species rarefaction curves (*MaoTau*) with 95% confidence intervals were scaled at the transect level (three transects per site) using EstimateS (version 9.1.0) (Colwell et al. 2004, 2012). The significant difference in species richness between the two sites was calculated using ANOVA, where each transect represented a data point. We calculated Bray Curtis dissimilarity of termite communities based on relative abundance data in R (version 1.2.5019) using the *vegdist* function in the [vegan] package, to examine the difference in species composition between and within the two sites. ANOVA was used to examine whether the intra-site Bray Curtis dissimilarities were statistically different between sites. Simpson’s inverse diversity index was calculated in EstimateS using transect data that were pooled for each site. Termite species density, i.e., the number of species recorded in each transect, and relative abundance, i.e., the number of species encountered in each microhabitat across each transect following Davies et al. (2003), were summed at the transect level. The data from the soil pits and the aboveground sampling were combined to create a dataset of termite richness and relative abundance for each transect. Termite species were allocated to feeding-groups according to Donovan et al. (2001) and Inward et al. (2007). Effect sizes, i.e., the standard deviations from the mean, were calculated using the formula

$$\begin{array}{l} \frac{\mu 1-\mu 2}{\sigma } \end{array}$$

where μ is the population mean and σ is the mean variation across both groups. The difference in feeding-group density and relative abundance between sites were examined using ANOVA and visualized in R *errbar* [Hmisc]. The difference in environmental variables, including leaf- and woody litter and canopy cover, between sites was examined using ANOVA. The impact of environmental variables on termite feeding-group density and relative abundance was analyzed using linear models and model selection was performed with *AICtab* [bbmle].

## Results

In this study, 57% of the sampled termite genera had never previously been recorded in Ecuador. Additionally, when comparing the large number of soldierless Apicotermitinae species recorded in this study (21 species) with the species list in Bahder et al. (2009) it was revealed that at least 15 soldierless species (Apicotermitinae) were newly recorded in Ecuador (Supp Fig. S2; Supp Table S1 [online only]).

**Fig. 1.**
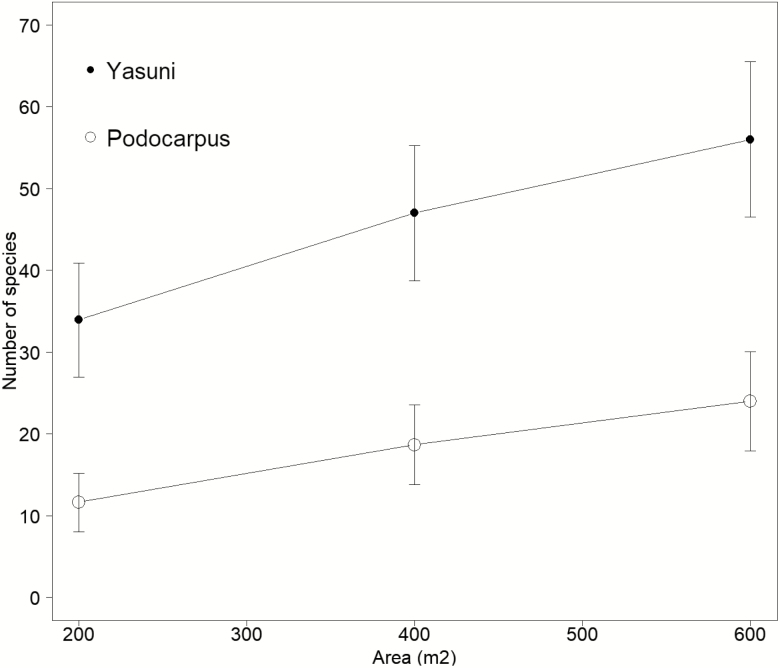
Termite species rarefaction curves (*Mao-Tau*) with 95% confidence intervals for each transect in Yasuní and Podocarpus. Three transects were sampled in each study site and the *x*-axis shows the accumulated sampling area (each transect covers 200 m^2^).

A total of 23 termite genera and 67 morphospecies were recorded in Yasuní and Podocarpus. Yasuní had significantly higher species richness (56 species in total, 32.7 ± 1.5 (± SD) species per transect) compared with Podocarpus (24 species in total, 11.0 ± 3.6 species per transect) (ANOVA, *P* < 0.01; df = 1,4; *F*-value = 85.40) (Fig. 1, Supp Fig. S2, Supp Table S1 [online only]). The intra-site Bray Curtis species dissimilarity index was similar in Yasuní (mean 0.42 ± 0.07) and Podocarpus (mean 0.42 ± 0.03), where 1 is complete dissimilarity and 0 is complete similarity (ANOVA, *P* < 0.05; df = 1,4; *F*-value = 0.00). The two sites shared 13 species and their average inter-site Bray Curtis dissimilarity index was 0.87 ± 0.01. The inverse Simpson’s diversity index for each site was 15.6 for Yasuní and 3.0 for Podocarpus, where species diversity increases with increasing index value.

**Fig. 2.**
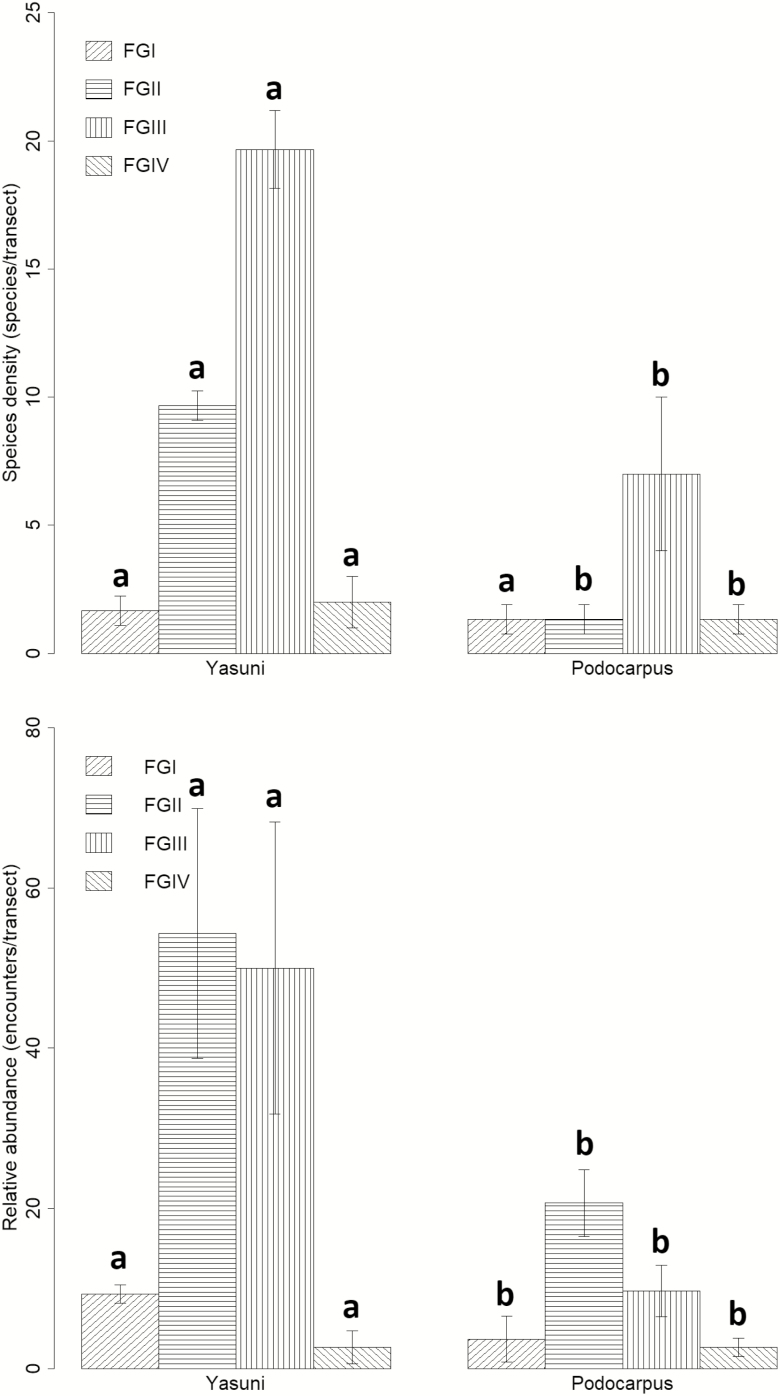
Termite species (A) density and (B) relative abundance (±SD) for feeding-groups in Yasuní and Podocarpus. Different letters represent significant differences among feeding-groups between the two sites. FGI = termites feeding on sound wood, FGII = termites feeding on wood and leaf litter, FGIII = termites feeding on organic-rich soil and humus with visible plant structure, FGIV = termites feeding on mineral soil with no visible plant structure.

Species densities of wood and litter-feeding (FGII) and humus-feeding (FGIII) termites were higher in Yasuní than in Podocarpus (ANOVA, FGII *P* < 0.001; df = 1,4; *F*-value = 312.50, FGIII *P* < 0.01, df = 1,4; *F*-value = 42.47), while no statistical difference was found for wood- (FGI) and soil-feeding (FGIV) termites between sites (ANOVA, FGI *P* > 0.05; df = 1,4; *F*-value = 0.50, FGIV *P* > 0.05; df = 1,4; *F*-value = 1.00) (Fig. 2). Although species densities of wood- (FGI) and soil-feeding (FGIV) termites were not statistically significant between the two sites, the effect sizes were medium to high (effect size: FGI = 0.58, FGIV = 0.85). Further, relative abundances of wood-feeding (FGI), wood- and litter-feeding (FGII), and humus-feeding (FGIII) termites were higher in Yasuní than in Podocarpus (ANOVA, FGI *P* < 0.05; df = 1,4; *F*-value = 9.97, FGII *P* < 0.05; df = 1,4; *F*-value = 13.10, FGIII *P* < 0.05; df = 1,4; *F*-value = 14.21), while no significant difference was found for soil-feeding (FGIV) termites between the two sites (ANOVA, FGIV *P* > 0.05; df = 1,4; *F*-value = 0.00) (Fig. 2).

There was no significant difference for either of the environmental variables between the two sites, although the effect sizes for all were large (effect size: canopy cover = 1.01, woody litter = 2.07, leaf litter = 1.40). However, when all data were pooled canopy cover had a significantly positive association with the species density of wood-feeding termites (FGI) and the relative abundance of wood- and litter-feeding termites (FGII) (ANOVA lm, FGI *P* < 0.05; df = 1,4; *F*-value = 31.85, FGII *P* < 0.05; df = 1,4; *F*-value = 30.64).

## Discussion

### Termite Species Richness and Composition

The majority of genera in this study were newly recorded in Ecuador along with at least 15 species of the soldierless Apicotermitinae. However, the lack of officially described termites, particularly soldierless species, makes comparisons difficult. No enteric valve descriptions (used to distinguish between soldierless species) were available in Bahder et al. (2009), which means that the five morphospecies recorded in that study are incomparable. To avoid this for future comparisons, we have provided photos of the enteric values of all Apicotermitinae morphospecies from this study (Supp Fig. S2).

The rarefaction curves suggest that sampling was not saturated in either of the study sites; however, based on data from Dahlsjö et al. (2014c), doubling the number of transects (from three to six) in each site would have resulted in a 5% increase in species richness, equivalent to three additional species in Yasuní and one additional species in Podocarpus. Termite species are therefore still to be discovered in Ecuador, and the described differences between the two sites are likely to be underestimations.

As predicted (Palin et al. 2010), species richness was lower in the mid-elevation site, Podocarpus, than in the lowland site, Yasuní. The decline in species richness (66% reduction from mid-elevation to lowland) was consistent with the drop in species numbers in other elevational studies which has been shown to vary between 55 and 70% over the same elevational range (Gathorne-Hardy et al. 2001, Palin et al. 2010). As termite elevation specialists do not exist, the termite population in Podocarpus was expected to represent a sub-set of the termite population in Yasuní (Jones et al. 2003, Palin et al. 2010). While the two sites shared 13 species, the inter-site comparisons showed almost complete dissimilarity between sites. Termite assemblage structures have been showed to be consistent over distances of 100–300 km (Palin et al. 2010, Bourguignon et al. 2011); however, as Yasuní and Podocarpus are located 700 km apart, the two forests are likely to have had access to different species pools which may have contributed to the high levels of dissimilarity. Studies that explore beta diversity of termites in South America are encouraged for better understanding of termite species boundaries (but see Cancello et al. (2014) for an account of termite species richness along a latitudinal gradient).

### Termite Feeding-Group Diversity

We predicted that the difference in feeding-group species density between sites would mainly be seen in soil-dwelling (FGIII and FGIV) and foraging (FGII) species due to their decline with elevation. While species density of only litter-feeding (FGII) termites and humus-feeding termites (FGIII) were significantly different between sites, the effect sizes for wood- (FGI) and soil-feeding (FGIV) termites were medium and large, respectively. The effect sizes suggest that species density of all feeding-groups was practically higher in Yasuní than in Podocarpus albeit not all significant. Additionally, the relative abundance of all feeding-groups with the exception of soil-feeding (FGIV) species was significantly higher in Yasuní than in Podocarpus.

*Cylindrotermes macrognathus* was the only FGII-feeding species that was recorded in Podocarpus and made up 56% of the total relative abundance in the site. Foraging species, such as FGII, are sensitive to elevation as the drop in ambient temperature slows the metabolic rate which affects termites that feed on low energy substrates, such as soil, or expend energy through foraging activities (Eggleton et al. 1998, Gathorne-Hardy et al. 2001, Nalepa 2011, Dahlsjö et al. 2015). While temperature was not recorded during sampling, the difference in ambient temperature between sites is approximately 8°C, which is likely to have a major impact on the ability of termites to thrive. However, *C. macrognathus* was the only recorded FGII species in Podocarpus, which may have enabled their abundance to surge due to the low competition for resources.

Soil- and humus-feeding species are particularly affected by reductions in metabolic rate due to the low energy content in soil compared with the relatively high energy content in wood. The non-significant difference in soil-feeding termites between sites contradicts this notion; however, this is most likely due to the overall low species density and abundance across the two sites. Additionally, although humus-feeding (FGIII) termites had significantly different species density and relative abundance between the two sites, they dominated over the soil-feeding (FGIV) termites. The difference between the soil—and humus-feeding termites may be due to the ability of humus-feeding species, such as *Anoplotermes banksi* and *Embiratermes neotenicus,* to thrive in a variety of conditions due to their construction of protective mounds (Constantino 1992, Martius and Ribeiro 1997, Korb 2011). The competition for soil resources may also have affected the relative abundance of soil-feeding (FGIV) termites as stable isotopes of the two group’s respective diets have been shown to overlap (Bourguignon et al. 2009).

While there was a difference in wood-feeding (FGI) relative abundance between the two sites only two species were recorded in Podocarpus and Yasuní. FGI species feed and nest in larger pieces of deadwood (one-piece nesters, see e.g., Higashi et al. 1992) and were therefore infrequently encountered as the searching activity focused on dead organic matter from ground level to breast height. More focused sampling, like the one in Bahder et al. (2009), is normally a more reliable method for recording FGI species as time is needed to find termites deep inside large pieces of wood often suspended several meters off the ground.

Tree diversity, particularly the presence of broad-leaved species, has been shown to increase the diversity of soil biota due to the increase in microhabitats and resource availability (Korboulewsky et al. 2016). Therefore, the difference in termite feeding-group relative abundance between the two study sites may have been linked to the higher number of tree species in Yasuní (Pitman et al. 2002, Lozano et al. 2010). Although, the association between leaf litter and termite abundance was not significant.

### Environmental Variables

In this study, we recorded three environmental variables including woody litter, leaf litter, and canopy cover due to the known effects of canopy disturbance and resource availability on termite diversity (Jones 2000). The variables were not statistically different between sites although the high effect sizes suggest that there was a practical difference. Of the environmental variables recorded in this study, canopy cover was the only significant driver of termite diversity when location was not taken into account. Canopy cover provides a more stable environment with little variation in temperature and moisture mainly associated with the impact on soil-feeding species. In this study, however, we only found an effect of canopy cover on wood- (FGI) and wood- and litter-feeding (FGII) termites. The low number of environmental variables is likely to have limited this analysis. Above we mention the effects of temperature on termite populations and its impact on metabolic rates. Soil chemical composition is also understood to be of high importance as soil pH above 5 and heightened levels of elements such as zinc, copper, magnesium and calcium have also been shown to cause a decline in termite diversity (Jones et al. 2010).

### Conclusions

In this study, we present termite genera and species that had previously never been scientifically recorded in Ecuador with more species expected to be encountered. Efforts to ensure that such data are comparable among sites to enable wider usage is encouraged. High taxonomic resolution is important for better understanding of beta and gamma diversity which have not been fully explored in South America due to the lack of data. Additionally, data on feeding-group diversity in a range of habitats will enable better understanding of ecosystem functioning as different termite feeding-groups inhabit different niches (Bignell and Eggleton 2000). Based on the data in this study it may be concluded that nutrient cycling in Yasuní is higher than that in Podocarpus due to the higher number of species and relative abundance. However, decomposition experiments over a larger range of sites are needed before any detailed conclusions can be made. Better understanding of forest ecosystems, their differences and similarities, and the availability of comparable data are paramount if we are to promote their conservation in a time of unprecedented deforestation.

## Supplementary Material

iez129_suppl_Supplementary-Figure_S1

iez129_suppl_Supplementary-Figure_S2

iez129_suppl_Supplementary-Table_S1
