# Supplementary figures and images for: Termite Diversity in Ecuador: A Comparison of Two Primary Forest National Parks
Source: J Insect Sci. 2020 Jan 9;20(1):4. doi: 10.1093/jisesa/iez129 (PMC6950023; doi:10.1093/jisesa/iez129)

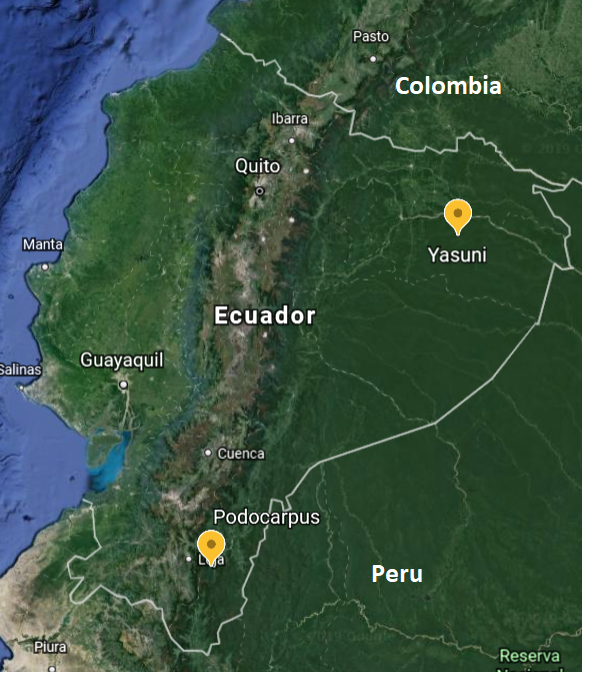

Supplement: iez129_suppl_Supplementary-Figure_S1 [file iez129_suppl_supplementary-figure_s1.png]

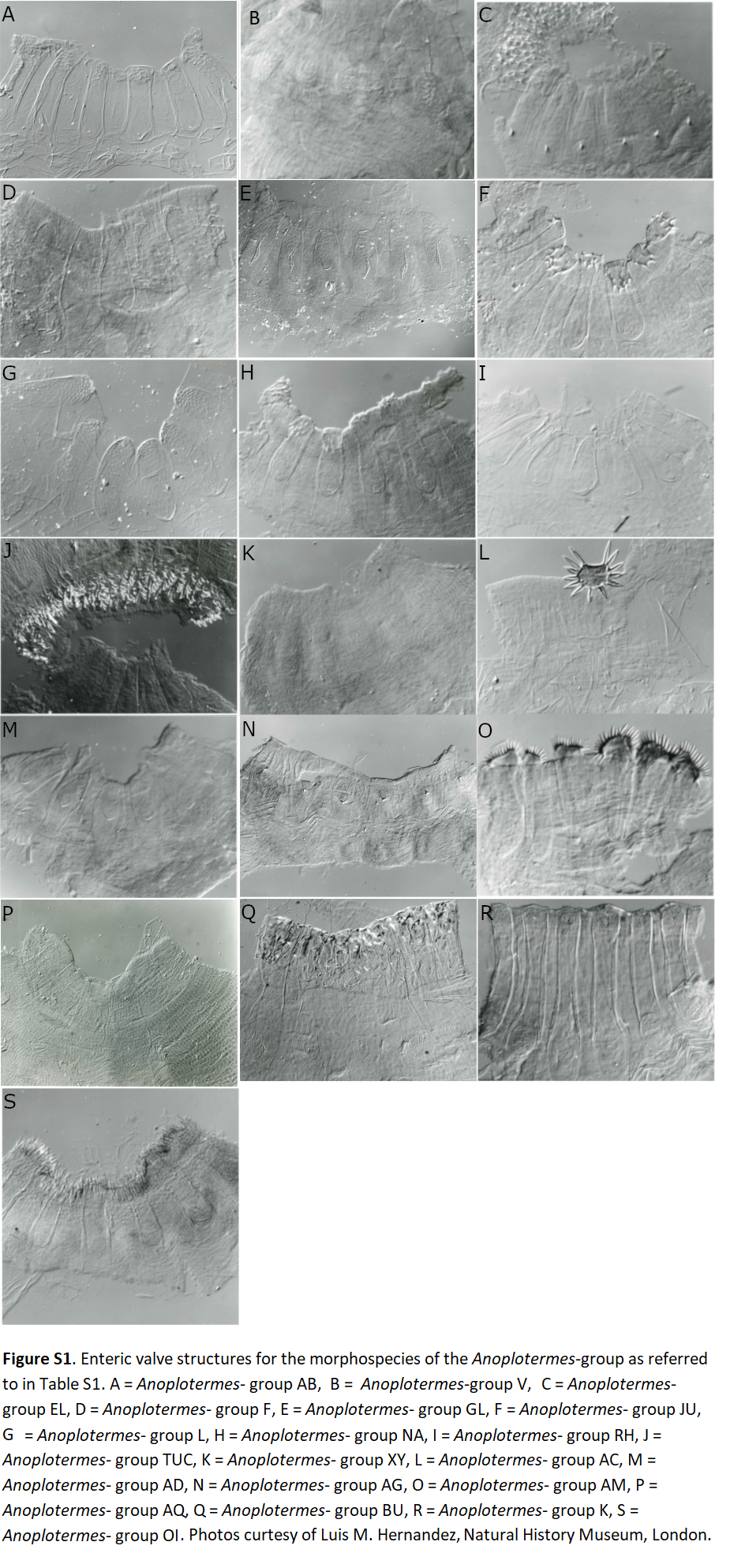

Supplement: iez129_suppl_Supplementary-Figure_S2 [file iez129_suppl_supplementary-figure_s2.png]
