# Supplementary material for: Termite Diversity in Ecuador: A Comparison of Two Primary Forest National Parks
Source: J Insect Sci. 2020 Jan 9;20(1):4. doi: 10.1093/jisesa/iez129 (PMC6950023; doi:10.1093/jisesa/iez129)
Supplement: iez129_suppl_Supplementary-Table_S1 [file iez129_suppl_supplementary-table_s1.pdf]

Table S1. Species and morphospecies from Yasuní National Park and Podocarpus National Park. Relative termite species abundance is provided for each of the three transects in each study site. Feeding-groups include: I = wood feeding non-Termitidae, II = wood-feeding Termitidae, III = humus-feeding Termitidae, IV = soil-feeding Termitidae. (encounters per macrohabitat per transect)

		Relative species abundance (encounters per microhabitat per transect)					
Taxa	Feeding-group	Yasuní			Podocarpus		
	Transect	1	2	3	1	2	3
RHINOTERMITIDAE							
RHINOTERMITINAE							
Dolichorhinotermes lanciarius	I	0	0	0	7	1	2
Dolichorhinotermes sp *I*	I	0	0	1	0	0	0
Rhinotermes sp *I*	I	1	0	0	0	0	0
HETEROTERMITINAE							
Heterotermes sp *I*	I	9	10	7	0	1	0
TERMITIDAE							
TERMITINAE							
Cylindrotermes flangiatus	II	18	17	15	0	0	0
Cylindrotermes macrognathus	II	2	0	0	22	24	16

Cylindrotermes sapiranga	II	9	3	16	0	0	0
Neocapritermes talpa	II	0	2	0	0	0	0
Neocapritermes talpoides	II	2	0	1	0	0	0
Crepititermes verruculosus	III	0	1	0	0	0	0
Cornicapritermes sp *I*	III	0	0	0	0	0	2
Termes sp *I*	III	0	0	1	0	0	0
SYNTERMITINAE							
Cyrilliotermes sp *I*	III	0	0	1	0	0	0
Embiratermes neotenicus	III	0	1	0	2	1	0
Embiratermes sp *I*	III	1	1	0	0	0	2
Embiratermes sp *IV*	III	0	1	0	0	0	0
APICOTERMITINAE							
Amplucrutermes inflatus	III	0	1	0	0	1	0
Anoplotermes banksi	III	4	6	1	0	1	0
Anoplotermes grp *AD*	III	0	0	0	1	0	0
Anoplotermes grp *AG*	III	0	0	0	0	0	3

Anoplotermes grp *EL*	III	0	0	0	0	2	0
Anoplotermes grp *GL*	III	0	0	1	0	0	0
Anoplotermes grp *JU*	III	1	0	0	0	0	0
Anoplotermes grp *L*	III	0	0	0	0	0	1
Anoplotermes grp *NA*	III	0	0	0	1	0	0
Anoplotermes grp *RH*	III	1	2	1	0	0	0
Anoplotermes grp *TUC*	III	0	2	0	0	0	0
Anoplotermes sp *AB*	III	6	1	1	0	0	2
Anoplotermes sp *AQ*	III	0	0	0	0	1	0
Anoplotermes sp *BU*	III	6	0	2	0	0	0
Anoplotermes sp *F*	III	0	1	0	0	0	1
Anoplotermes sp *K*	III	1	3	0	0	0	0
Anoplotermes sp *V*	III	6	3	5	2	1	1
Anoplotermes sp *XY*	III	3	2	0	0	1	0
Aparatermes thornatus	III	0	0	1	0	0	0
Ruptitermes sp *A*	III	0	0	0	0	1	0
Hydrecotermes ariensho	III	1	0	0	0	1	0
Hydrecotermes kawaii	III	0	1	0	0	0	0

Anoplotermes grp *AC*	IV	1	0	0	0	0	0
Anoplotermes grp *AM*	IV	0	0	0	0	1	0
Anoplotermes grp *OI*	IV	0	1	0	0	0	0
Patawatermes nigripunctatus	IV	2	0	0	2	0	0
Humutermes krishnai	IV	2	1	1	2	2	2
NASUTITERMITINAE							
Atlantitermes snyderi	III	1	2	1	0	0	0
Atlantitermes sp *II*	III	0	0	1	0	0	0
Constrictotermes sp *I*	III	0	0	0	0	1	0
Ereymatermes sp *I*	III	2	4	2	0	0	0
Nasutitermes sp *I*	II	0	0	1	0	0	0
Nasutitermes sp *II*	II	0	1	0	0	0	0
Nasutitermes sp *III*	II	1	0	1	0	0	0
Nasutitermes sp *IV*	II	0	0	4	0	0	0
Nasutitermes sp *V*	II	0	1	0	0	0	0
Nasutitermes sp *VI*	II	5	5	15	0	0	0
Nasutitermes sp *VII*	II	9	5	5	0	0	0

Nasutitermes sp *VIII*	II	5	2	7	0	0	0
Nasutitermes sp *X*	II	0	1	0	0	0	0
Nasutitermes sp *XI*	II	1	0	4	0	0	0
Nasutitermes sp *XII*	II	5	3	0	0	0	0
Nasutitermes sp *XIII*	II	0	0	1	0	0	0
Nasutitermes sp *XIV*	II	1	0	0	0	0	0
Silvestritermes sp *I*	III	1	0	0	0	0	0
Silvestritermes sp *II*	III	15	24	4	0	0	0
Silvestritermes sp *III*	III	1	2	2	0	0	0
Silvestritermes sp *IV*	III	2	1	1	0	0	0
Subulitermes sp *I*	III	2	1	0	0	0	0
Subulitermes sp *II*	III	1	0	1	0	0	0
Triangularitermes sp *II*	III	2	0	2	0	0	0
